# Modeling microRNA-mRNA Interactions Using PLS Regression in Human Colon Cancer

**DOI:** 10.1186/1755-8794-4-44

**Published:** 2011-05-19

**Authors:** Xiaohong Li, Ryan Gill, Nigel GF Cooper, Jae Keun Yoo, Susmita Datta

**Affiliations:** 1 Department of Bioinformatics and Biostatistics, University of Louisville, (485 E. Gray St.), Louisville, KY 40202, USA; 2 Department of Anatomical Sciences and Neurobiology, University of Louisville, (500 S. Preston St.), Louisville, KY 40202, USA; 3 Department of Mathematics, University of Louisville, (328 Natural Sciences Building), Louisville, KY 40292, USA; 4 Department of Statistics, Ewha Womans University, (52 Ewhayeodae-gil, Seodaemun-gu), Seoul, 120-750, Republic of Korea

## Abstract

**Background:**

Changes in microRNA (miRNA) expression patterns have been extensively characterized in several cancers, including human colon cancer. However, how these miRNAs and their putative mRNA targets contribute to the etiology of cancer is poorly understood. In this work, a bioinformatics computational approach with miRNA and mRNA expression data was used to identify the putative targets of miRNAs and to construct association networks between miRNAs and mRNAs to gain some insights into the underlined molecular mechanisms of human colon cancer.

**Method:**

The miRNA and mRNA microarray expression profiles from the same tissues including 7 human colon tumor tissues and 4 normal tissues, collected by the Broad Institute, were used to identify significant associations between miRNA and mRNA. We applied the partial least square (PLS) regression method and bootstrap based statistical tests to the joint expression profiles of differentially expressed miRNAs and mRNAs. From this analysis, we predicted putative miRNA targets and association networks between miRNAs and mRNAs. Pathway analysis was employed to identify biological processes related to these miRNAs and their associated predicted mRNA targets.

**Results:**

Most significantly associated up-regulated mRNAs with a down-regulated miRNA identified by the proposed methodology were considered to be the miRNA targets. On average, approximately 16.5% and 11.0% of targets predicted by this approach were also predicted as targets by the common prediction algorithms TargetScan and miRanda, respectively. We demonstrated that our method detects more targets than a simple correlation based association. Integrative mRNA:miRNA predictive networks from our analysis were constructed with the aid of Cytoscape software. Pathway analysis validated the miRNAs through their predicted targets that may be involved in cancer-associated biological networks.

**Conclusion:**

We have identified an alternative bioinformatics approach for predicting miRNA targets in human colon cancer and for reverse engineering the miRNA:mRNA network using inversely related mRNA and miRNA joint expression profiles. We demonstrated the superiority of our predictive method compared to the correlation based target prediction algorithm through a simulation study. We anticipate that the unique miRNA targets predicted by the proposed method will advance the understanding of the molecular mechanism of colon cancer and will suggest novel therapeutic targets after further experimental validations.

## Background

Colon cancer is the third most common cancer in the United States [1] and contributes to over 655,000 deaths per year worldwide. However when diagnosed early, it is one of the most treatable cancers [2]. Many efforts are focused on detection rates and screening utilization [3,4]. The primary treatment for colon cancer may involve surgery, chemotherapy, biological therapy or radiation [5]. Unfortunately, these treatments often damage normal cells and tissues, so side effects are common as are the possibilities of drug resistance and disease recurrence [6]. Therefore, the identification of new biomarkers for early diagnosis and prognosis of human colon cancer is of great interest. Furthermore, such biomarkers might be useful for the development of novel therapeutics.

Recently miRNAs have been suggested to be among these potential biomarkers [7]. The miRNAs are short (~21nt) non-coding RNAs that regulate gene expression by causing transcript degradation or translational repression [8,9]. In animals, they have been found to regulate a wide range of biological processes such as stem cell maintenance, development, metabolism, host-viral interaction, differentiation, proliferation and apoptosis [10]. Their activities are also implicated in cancer progression or suppression by affecting oncogenesis, growth, invasion and metastasis [11]. Studies suggest that three of these miRNAs, miR-15a, miR-16 [12,13] and let-7 [14,15] can function as tumor suppressors, while miR-155 and miR-21 play roles in oncogenesis [16,17]. Many studies have also shown that miRNAs play a critical role in cancer initiation and progression. For example, miRNA-135a and miR-135b are reported to be involved in the initiation of human colon cancer by targeting the adenomatous polyposis coli gene (APC) [18]. Alterations in miRNA expression patterns are commonly associated with numerous human cancers, such as colorectal cancer and chronic lymphocytic leukemia [19]. Lu et al. used 218 miRNAs expression profiles from 334 samples, including multiple human cancers, to obtain information on the developmental lineage and differentiation state of the tumors [20]. They found most miRNAs in tumors were down-regulated compared to expression levels in normal tissues. Thus, miRNA expression profiles may be a useful tool in human cancer diagnosis and in designing treatments strategies. Nevertheless, specific targets of the abnormally expressed miRNAs and their biological functions remain largely unknown. So, accurately identifying those genes regulated by such miRNAs and their biological functions in affecting cancer development and progression is of utmost importance.

Although, an increasing number of miRNA targets have been validated experimentally http://diana.cslab.ece.ntua.gr/tarbase, a majority of miRNA targets are currently unknown and bioinformatics algorithms remain the primary means of predicting these putative targets. The principles of these algorithms are based on sequence complementarity, species conservation, thermodynamic stability, and site accessibility. Currently, there are eight widely used algorithms for miRNA target prediction (DIANA-microT 3.0, EIMMo, miRanda, miRBase, PicTar, PITA, RNA22 and TargetScan 5.1) [11]. However, the utility of these computational techniques is limited by various factors including miRNA size, identification of 3´ UTR and conservation analysis [19]. Furthermore, to our knowledge, none of these algorithms incorporate high-throughput microarray gene expression profiling data to predict miRNA targets.

As many miRNAs initiate the degradation of target mRNA transcripts [8], simultaneous expression profiles of miRNAs and mRNAs should reveal the existence of such inverse relationships. Huang et al. in 2007 was the first to utilize paired expression profiles of miRNAs and mRNAs to identify functional miRNA targets using a Bayesian data analysis algorithm (GeneMiR++) [21]. More recently, a similar strategy was used to identify hepatitis C virus (HCV)-associated miRNA-mRNA regulatory modules [22]. However, the integrative computational method used in that paper was based on the similarities in the expression profiles of miRNAs and mRNAs across samples to calculate a simple miRNA-mRNA correlation matrix. This pairwise correlation method may grossly undermine the existing collinearities amongst the covariates. The complex interrelationship of multiple miRNAs influencing the expression of one mRNA can be modeled by a regression method. However, due to the large number of miRNAs compared to the sample size and the existing collinearities amongst the covariates (miRNAs) it is impossible to use an ordinary linear regression model. So we use PLS regression model to cope with these issues. Associations between gene expression profiles have been previously studied using a PLS regression approach in [23]. Since then several others have proposed using PLS in the microarray and proteomics contexts [24-27]. Standard linear regression is not applicable for microarray gene expression data where the number of covariates far exceeds the sample size. PLS is a method for constructing predictive models when there are numerous covariates and many of them are highly collinear. To the best of our knowledge, a PLS regression method has not been used previously to integrate miRNA and mRNA microarray data to predict miRNA targets.

Thus, the PLS regression method was used to explore the likely associations/interactions between miRNA and mRNA using expression data previously collected from 7 colon tumors [20]. We have used Pathway analysis to understand the biological processes linked to these miRNAs and their predicted target mRNAs in the context of colorectal cancer.

## Results

### Outline of our analysis

**Figure 1:**
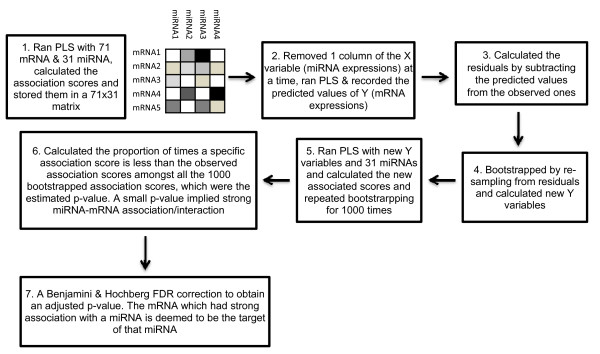
**Algorithm to find miRNA targets**. Illustrated is a workflow involving PLS and statistical testing to find the significant mRNA targets for each down-regulated miRNA.

We used a multi-step approach in our analysis. In STEP 1, an un-paired t-test was used with FDR correction methods to identify differentially expressed miRNAs and mRNAs in colon tumors and normal colon tissues. In STEP 2, the PLS regression method and statistical tests on the association measures were performed. Repeated bootstrap samples were used to identify significant associations/interactions between miRNA and mRNA using 31 down-regulated miRNAs and 71 up-regulated mRNAs. This PLS-bootstrap algorithm work-flow is outlined in the Figure 1. In STEP 3, we compared the predictions from our PLS regression method with those from TargetScan 5.1 and miRanda. In STEP 4, a gene ontology analysis was constructed with the aid of Pathway Studio software. In STEP 5, miRNA-mRNA interacting networks were developed with the aid of Cytoscape 2.7 software. In STEP 6 and 7, IPA and IPA pathway designer were used to create a functional analysis of biological processes related to miRNAs and their target mRNAs. Finally, in STEP 8, a simulation was performed to evaluate the false detection rate for PLS. Steps 1-3 can be considered as the discoveries after mining the real data and Steps 6-8 correspond to the validities of these mined results.

### STEP 1. Differentially expressed miRNA and mRNA in human colon tissues

The miRNA and mRNA microarray expression profiles from the same tissues including 7 human colon tumor tissues and 4 normal tissues were analyzed with the aid of GeneSpring GX10 software (Agilent, CA). The Benjamini & Hochberg FDR (false discovery rate) was used to obtain adjusted p-values for unpaired t-tests [28]. Differentially expressed miRNAs and mRNAs with adjusted p-values less than or equal to 10% and the absolute fold changes greater than or equal to 1.2 were obtained. We found a total of 31 down-regulated miRNAs and 2 up-regulated miRNAs that fit these criteria (Additional file 1, table S1). In addition, we found 73 up-regulated mRNAs and 63 down-regulated mRNAs (Additional file 2, table S2). Some of the mRNAs did not have gene IDs. There were only two up-regulated miRNAs using the above mentioned cutoff. Therefore, in this study we selected the 31 down-regulated miRNAs and the 71 up-regulated mRNAs to find the most likely miRNA:mRNA negative/inverse associations with the aid of the PLS regression method. Among the differentially expressed miRNAs, only miR-135b and miR-221 are significantly up-regulated (4.3 and 1.7 fold, respectively) in the colon cancer tissue samples. The remaining 31 miRNAs (Additional file 1, table S1) were significantly down-regulated in these samples.

### STEP 2. miRNA:mRNA Associations predicted by PLS

To identify the most likely prospective targets the 31 down-regulated miRNAs were used as independent variables and the 71 mRNAs were denoted as dependent variables in the PLS regression analysis. Subsequently, statistical tests were performed to identify significant negative associations between each mRNA with the 31 down-regulated miRNAs. The algorithm flow diagram for this multi-step procedure is shown in Figure 1.

Table 1 lists the 31 down-regulated miRNAs and their associated target mRNAs predicted by the PLS regression method. The maximum number of predicted targets was 25 for miR-30d, and the minimum number of predicted targets was one for miR-1, miR-100 and rno-miR-140 (Table 1). The number of predicted targets for each member of the miR-30 family (a-e) was 22, 20, 15, 25 and 17, respectively. Table 2 lists the mRNAs for each of those miRNAs which have been previously reported to have associations with some form of cancer. Table 2 also contains some additional results related to STEP 3.

**Table 1:** Identification of 31 down-regulated miRNAs from a list of 71 up-regulated mRNAs

Down-regulated miRNA	miRNA family	No. of mRNA targets	Target genes predicted by the PLS method
*hsa-let-7 g	Let-7 g	17	BUD31, COTL1, CPNE1, EIF3B, FTL, GTF3C1, IFITM1, NAP1L4, NUDT1, PHB, PSMC2, PSMC4, PUF60, Ras-Like protein, RPL10, SRD5A3, WDR36

hsa-miR-1	miR-1	1	RPL10

hsa-miR-100	miR-100	1	GSTP1

*hsa-miR-101	miR-101	7	ATP6V0B, GTF2IRD1, GUK1, NEBL, PSMB4, UBE2I, VKORC1

hsa-miR-10b	miR-10b	2	MIF, PSMC2

hsa-miR-126	miR-126	16	ANXA2, CSTB, DDIT4, GPATCH3, GTF2IRD1, GUK1, MDK, NDK, NEBL, OAZ1, PSMB4, S100A11, SLC7A11, TBCD, UBA6, VKORC1

hsa-miR-130a	miR-130	2	GSTP1, RPL34

*hsa-miR-133a	miR-133	17	ANXA2, ATP6V0B, CSTB, GPATCH3, GPX1, GTF2IRD1, NAP1L4, NEBL, OAZ1, POLR2H, PSMB4, RPL10, SLC7A11, TBCD, UBA6, UBE2I, VKORC1

hsa-miR-142-5p	miR-142	6	ARPC1B, DDIT4, GSTP1, MDK, TUFM, USP22

*hsa-miR-150	miR-150	20	ABHD12, C7orf28A, CLIC1, CPNE1, EIF3B, GSTP1, GTF3C1, IFITM1, LAPTM5, NAP1L4, ODC1, PRDX2, PSMB2, PSMC4, PSMD8, PUF60, RPL10, SRD5A3, TBCD, Wilm's tumor-related protein

*hsa-miR-15a	miR-15	8	C7orf28A, CAPG, CPNE1, GTF3C1, NUDT1, PSMD8, WDR36, Wilm's tumor-related protein

*hsa-miR-16	miR-16	8	FTL, GTF3C1, LAPTM5, PHB, PSMD8, SRD5A3, WDR36, Wilm's tumor-related protein

hsa-miR-195	miR-195	2	PSMD8, RPL10

hsa-miR-26a	miR-26	3	CAPG, FTL, GTF3C1

hsa-miR-26b	miR-26	15	ATP6V0B, BUD31, CANX, CAPG, EIF3B, FTL, MIF, NAP1L4, NUDT1, ODC1, POLR2H, PSMC2, PSMC4, UBE2I, WDR36

hsa-miR-28	miR-28	21	ABHD12, ARPC1B, CLIC1, CPNE1, EIF3B, FTL, GTF3C1, IFITM1, LAPTM5, NAP1L4, ODC1, PRDX2, PSMB2, PSMC4, PSMD8, PUF60, RPL10, RPS9, SRD5A3, TUFM, Wilm's tumor-related protein

*hsa-miR-29b	miR-29	15	ANXA2, CDC42SE1, CSTB, DDIT4, GNPTG, GPATCH3, GPX1, ITPA, MDK, PSMB4, S100A11, SLC7A11, USP22, VKORC1, ZNF37A

*hsa-miR-29c	miR-29	7	ABHD12, C7orf28A, PSMD8, RPL10, TBCD, USP22, ZNF37A

*hsa-miR-30a	miR-30	22	ANXA2, C7orf28A, CAPG, CSTB, DAP, GPATCH3, GPX1, GTF2IRD1, GUK1, ITPA, MMP11, NEBL, NUDT1, OAZ1, POLR2H, PSMB4, SLC7A11, TBCD, UBA6, UBE2I, VKORC1, ZNF37A

*hsa-miR-30b	miR-30	20	ANXA2, CDC42SE1, CSTB, DDIT4, GNPTG, GPATCH3, GPX1, GPX4, GSTP1, GTF2IRD1, ITPA, MDK, NDK, OAZ1, PSMB4, S100A11, SLC7A11, USP22, VKORC1, ZNF37A

*hsa-miR-30c	miR-30	15	ANXA2, CSTB, GPATCH3, GSTP1, GTF2IRD1, ITPA, MDK, NDK, OAZ1, PSMB4, S100A11, SLC7A11, UBA6, VKORC1, ZNF37A

*hsa-miR-30d	miR-30	25	ANXA2, ATP6V0B, BHLHE40, C7orf28A, CAPG, CDC42SE1, CSTB, DAP, DDIT4, GPATCH3, GPX1, GTF2IRD1, MIF, MMP11, NEBL, POLR2H, PSMB4, RPL10, S100A11, SLC7A11, TBCD, UBA6, UBE2I, VKORC1, ZNF37A

*hsa-miR-30e	miR-30	17	ATP6V0B, BHLHE40, C7orf28A, CAPG, CSTB, DAP, GPATCH3, GPX1, MMP11, NEBL, NUDT1, OAZ1, POLR2H, RPL10, TBCD, VKORC1, ZNF37A

hsa-miR-32	miR-32	5	C7orf28A, ODC1, PSMD8, RPL10, TBCD

*hsa-miR-34b	miR-34	12	BUD31, CANX, EIF3B, FTL, GTF3C1, IFITM1, PHB, PSMC4, PUF60, RPL34, SRD5A3, WDR36

hsa-miR-99a	miR-99	19	ANXA2, CSTB, DDIT4, GPATCH3, GSTP1, GTF2IRD1, MDK, MIF, NDK, NEBL, OAZ1, POLR2H, S100A11, SLC7A11, TBCD, UBA6, UBE2I, VKORC1, ZNF37A

mmu-miR-10b	miR-10	2	Ras-Like protein, WDR36

mmu-miR-151	miR-151	13	ATP6V0B, BHLHE40, C7orf28A, CAPG, GTF2IRD1, MIF, MMP11, NUDT1, OAZ1, POLR2H, RPL10, TBCD, VKORC1

*mmu-miR-342	miR-342	21	ABHD12, ARPC1B, CLIC1, CPNE1, EIF3B, FTL, GTF3C1, IFITM1, NAP1L4, ODC1, PSMB2, PSMC4, PSMD8, PUF60, RPL10, RPL34, RPS9, SRD5A3, TGFBI, TUFM, Wilm's tumor-related protein

rno-miR-140	miR-140	1	NDK

rno-miR-151	miR-151	16	ANXA2, CDC42SE1, CSTB, DDIT4, GNPTG, GPATCH3, GPX1, GTF2IRD1, ITPA, MDK, MMP11, PSMB4, S100A11, SLC7A11, USP22, ZNF37A

*miRNAs are selected as cancer-mediators based on the literature.

**Table 2:** Identification of miRNA targets from 15 down-regulated and 71 up-regulated mRNAs

Cancer-related and down-regulated miRNA	miRNA family	No. of target mRNAs	No. (%) of overlapping PLS predicted mRNAs with TargetScan5.1	No. (%) of overlapping PLS predicted mRNAs with miRanda
hsa-let-7 g	Let-7 g	16	0	2(12.5)

hsa-miR-101	miR-101	7	0	0

hsa-miR-133a	miR-133	17	3(17.6)	2(11.8)

hsa-miR-150	miR-150	20	3(15.0)	1(5.0)

hsa-miR-15a	miR-15	8	1(12.5)	1(12.5)

hsa-miR-16	miR-16	8	1(12.5)	0

hsa-miR-29b	miR-29	15	3(20.0)	2(13.3)

hsa-miR-29c	miR-29	7	2(28.6)	1(14.3)

hsa-miR-30a	miR-30	22	3(13.6)	3(13.6)

hsa-miR-30b	miR-30	20	4(20.0)	2(10.0)

hsa-miR-30c	miR-30	15	1(6.7)	1(6.7)

hsa-miR-30d	miR-30	25	7(28.0)	5(20.0)

hsa-miR-30e	miR-30	17	3(17.6)	3(17.6)

hsa-miR-34b	miR-34	12	3(25.0)	0

mmu-miR-342	miR-342	21	4(19.0)	1(4.8)

**Total**	-	-	**16.5**	**11.0**

### STEP 3. Comparisons between PLS Regression, TargetScan 5.1 and miRanda

Comparing the PLS regression method with other computational miRNA-target prediction algorithms such as TargetScan 5.1 and miRanda we found some areas of agreement. Thus, some of the miRNA-targets predicted by PLS were also predicted by either TargetScan or miRanda or both of them (Table 2). This table provides the number of miRNA-targets for those miRNAs reportedly linked to cancer [29-42]. It shows the percentage of common targets predicted by our use of the PLS regression method, with TargetScan 5.1 and miRanda.

Using 15 down-regulated miRNAs (let-7 g, miR-101, miR-133a, miR-150, miR-15a, miR-16, miR-29b, miR-29c, miR-30a, miR-30b, miR-30c, miR-30d, miR-30e, miR-34b and miR-342), known to be associated with cancer, we found 16.5% and 11.0% of our PLS-predicted miRNA-targets, on average, were also predicted as targets for the corresponding miRNAs by TargetScan5.1 and miRanda, respectively (Table 2). Moreover, we found that five out of 25 of our PLS-predicted targets (20%) for miR-30d were also predicted as targets for this miRNA by both TargetScan5.1 and miRanda. These five target genes predicted by our PLS regression approach, TargetScan 5.1 and miRanda were BHLHE40, DDIT4, NEBL, SLC7A11 and UBE2I. In this regard, it is interesting to note that our method identified more common targets with TargetScan than miRanda. From Tables 1 and 2 it is evident that our analysis provides many novel targets that were not identified by these existing algorithms which may prove to be significant biomarkers for colon cancer. In order to further pursue the integrative analysis an association network for these significantly associated miRNAs and mRNAs was constructed (see STEP 5).

### STEP 4. Gene ontology (GO) terms for miRNA-targets in the category of biological processes

To gain further insight into the biological functions of each miRNA and its predicted targets, the 71 up-regulated mRNAs from the colon cancer microarray were first analyzed with the aid of Pathway Studio 7 (Ariadne Genomics Inc., Rockville, MD, USA). This analysis was used to obtain the gene ontological (GO) terms for the category of biological processes. We selected three of the most significant GO terms with all of the unadjusted p-values less than 0.02 except one function termed DNA replication which had a p-value 0.05. The corresponding adjusted p-values were less than 0.06 and 0.13 respectively. Almost all the terms are significant at FDR 10%. The remaining term is significant at FDR 13%. In Table 3 the significant GO terms associated with the predicted targets for 15 miRNAs linked to colorectal cancer tissues are listed as biological processes. The significant biological processes include anaphase-promoting complex (APC)-dependent proteasomal ubiquitin-dependent protein catabolic process, negative or positive regulation of ubiquitin-protein ligase activity during mitotic cell cycle, polyamine biosynthetic process, oxidative stress, oxidation reduction, oxidoreductase activity, glutathione peroxidase process, regulation of cell proliferation and apoptosis, translational elongation and lipid metabolic process. Interestingly, many of the target mRNAs expressed in the colorectal cancer tissues and associated with the miRNAs appear to be involved in protein degradation and apoptosis and are linked to the oxidative stress signal transduction pathway. Therefore, these data highlight the oxidative stress mechanism that is operative in colorectal cancer tissue.

**Table 3:** Gene ontology (GO) terms for miRNA-targets in the category of biological processes

Cancer-related and down-regulated miRNA	miRNA family	No. of mRNAs	GO terms
hsa-let-7 g	Let-7 g	16	Anaphase-promoting complex(APC)-dependent proteasomal ubiquitin-dependent protein catabolic process, response to oxidative stress and regulation of apoptosis

hsa-miR-101	miR-101	7	Ubiquitin-dependent protein catabolic process, APC-dependent proteasonmal ubiquitin-dependent protein catabolic process and oxidoreductase activity

hsa-miR-133a	miR-133	17	Polyamine biosynthetic process,glutathione peroxidase process, Ubiquitin-dependent protein catabolic process and regulation of apoptosis

hsa-miR-150	miR-150	20	Polyamine biosynthetic process, APC-dependent proteasomal ubiquitin-dependent protein catabolic process, Glutathione peroxidase activity

hsa-miR-15a	miR-15	8	Anaphase-promoting complex-dependent proteasomal ubiquitin-dependent protein catabolic process, response to oxidative stress and proteasome complex

hsa-miR-16	miR-16	8	APC-dependent proteasomal ubiquitin-dependent protein catabolic process, negative regulation of cell proliferation and oxidoreductase activity

hsa-miR-29b	miR-29	15	Regulation of cell proliferation, glutathione peroxidase activity and regulation of apoptosis

hsa-miR-29c	miR-29	7	Ubiquitin-dependent protein catabolic process, APC-dependent proteasomal ubiquitin-dependent protein catabolic process and translational elongation

hsa-miR-30a	miR-30	22	Polyamine biosynthetic process, ubiquitin-dependent protein catabolic process, and regulation of apoptosis

hsa-miR-30b	miR-30	20	Ubiquitin-dependent protein catabolic process, polyamine biosynthetic process and oxidative stress

hsa-miR-30c	miR-30	15	Polyamine biosynthetic process, ubiquitin-dependent protein catabolic process, and regulation of apoptosis

hsa-miR-30d	miR-30	25	Regulation of apoptosis, glutathione peroxidase activity and ubiquitin-dependent protein catabolic process

hsa-miR-30e	miR-30	17	Polyamine biosynthetic process, response to oxidative stress and regulation of apoptosis

hsa-miR-34b	miR-34	12	Regulation of cell proliferation, oxidoreductase activity and translational elongation

mmu-miR-342	miR-342	21	Polyamine biosynthetic process, translational elongation and APC-dependent proteasomal ubiquitin-dependent protein catabolic process

### STEP 5. Visualization of miRNA:mRNA association networks

**Figure 2:**
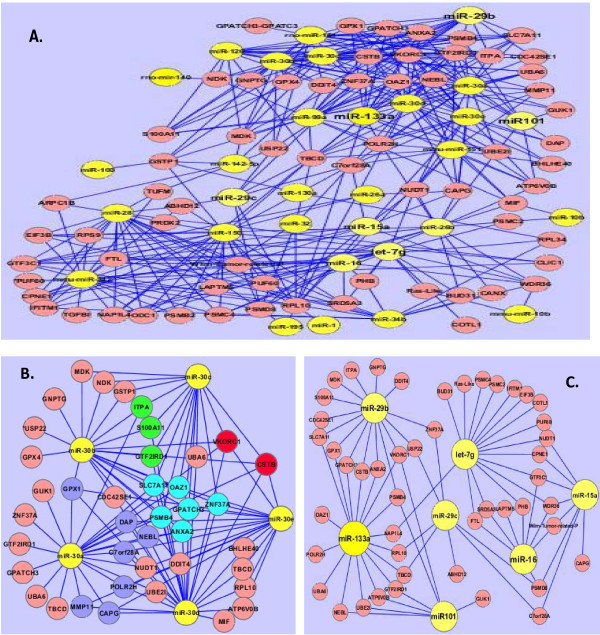
**miRNA-mRNA association network**. **A. **miRNA-mRNA interaction network with 97 nodes and 155 connections between 31 down-regulated miRNAs and 71 up-regulated mRNAs. miRNAs are in yellow while mRNAs are in pink. **B. **A sub-network depicting miRNA-mRNA interactions predicted from the miR-30 family. miRNAs are in yellow and mRNAs are in other colors. **C. **A sub-network depicting miRNA:mRNA interactions predicted from other cancer-associated miRNAs: let-7 g, miR-101, miR-133a, miR-15a, miR-16, miR-29b and miR-29c.

Using the statistically significant *β_ij _*-association scores between the i-th mRNA and j-th miRNA, derived from the PLS regression method, we constructed association networks including the significant miRNAs and mRNAs. The resulting miRNA:mRNA association network had 97 nodes and 155 connections between the 31 down-regulated miRNAs and the 71 up-regulated mRNAs (Figure 2A). We developed a network to demonstrate the overlapping miRNA targets for miRNAs in the miR-30 family (a-e) (Figure 2B) since these miRNAs have a large number of mRNA targets. The number of targets for miR-30 a-e was 22, 20, 15, 25 and 17, respectively (Table 3). This network (Figure 2B) indicated that two mRNAs (VKORC1 and CSTB; in red) were predicted to be targets of five miR-30 miRNAs, six mRNAs (ANXA2, GPATCH3, OAZ1, PSMB4, SLC7A11, ZNF37A; in turquoise) were targeted by four of the miRNAs, and ten mRNAs (CAPG, C7orf28A, DAP, GPX1, ITPA, MMP11, NEBL, POLR2H, S100A11 and GTF2IRD1; 3 in green and 7 in blue) were targeted by three of the miRNAs (Figure 2B). Networks were also developed for the seven miRNAs (let-7 g, miR-101, miR-133a, miR-15a, miR-16, miR-29b and miR-29c) closely related to cancer and their associated mRNAs (Figure 2C). Most of the targets mentioned here have been reported to be linked to cancer. For example, ANXA2 up-regulation was reported to be associated with human hepatocellular carcinoma, pancreatic adenocarcinoma, high-grade glioma, gastric carcinoma, acute promyelocytic leukemia and primary colorectal cancer [43]. Also, *CapG *message and protein are reported to be up-regulated in colorectal cancer [44].

### STEP 6. Pathway analysis with the aid of IPA

**Figure 3:**
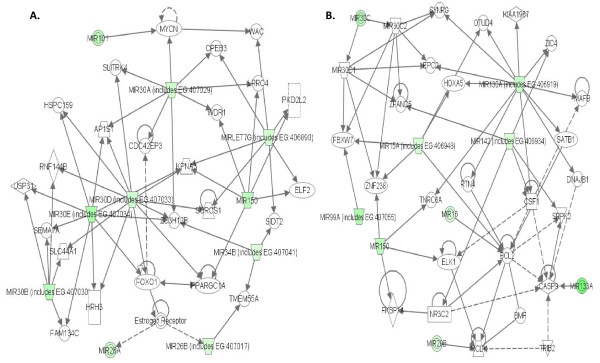
**Top miRNA networks**. **A. **miRNA-associated network with most statistically significant (top) functions related to cancer, reproductive system disease, and genetic disorders. The ten miRNAs, colored green were presented in the list of 31 down-regulated miRNAs (Table 1). The genes with white color are not from our data set and recruited by the IPA knowledge base. **B. **miRNA-associated network with top functions related to cell death, hematological system development and function, and cancer. The nine miRNAs, colored green were also present in the list of 31 down-regulated miRNAs.

To gain additional insight into prospective miRNA linked biological networks and canonical pathways, the 31 down-regulated miRNAs from the colon cancer microarrays were analyzed with the aid of Ingenuity Pathway Analysis (IPA) software. We focused on two miRNA-associated networks related to cancer. The most significant (top) functional pathways highlighted by IPA consisted of 1) cancer, reproductive system disease, and genetic disorders and 2) cell death, hematological system development and function, and cancer. We found that ten of the down-regulated miRNAs (miR101, miR26a, miR26b, miR30a, miR30b, miR30d, miR30e, miR34b, miR-let7 g and miRN140) were grouped together in a functional network (Figure 3A) and nine of the down-regulated miRNAs (miR-130a, miR-133a, miR-142, miR-150, miR15a, miR-16, miR-29b, miR-30c and miR-99a) were grouped together in a second network (Figure 3B). These are colored in green in Figures 3A and 3B.

In parallel, we used IPA to investigate the 71 up-regulated mRNAs. We were particularly interested in finding biological networks, functions and canonical pathways associated with these mRNAs that may be linked to human cancers. Therefore, we focused on the four most statistically significant networks with functions related to tumor morphology, cell signaling, immunological disease, cellular growth and proliferation (Table 4), functions of cancer (Table 5) and cancer-associated canonical pathways (Table 6). The second column of Table 4, 5 and 6 includes all genes associated with the top four networks, function, or canonical pathways of cancer which are also predicted by the PLS regression method as possible miRNA targets.

**Table 4:** 15 miRNAs and target genes involved in the cancer-related gene networks

Top Network	Target genes	Top functions	The nos. of miRNAs associated
Network 1	BHLHE40, CANX, CDC42SE1, GPX1, GPX4, GSTP1, MDK, MIF, MMP11, OAZ1, ODC1, PPIB, PRDX2, PSMA3, PSMB2, PSMB4, PSMC2, PSMC4, PSMD8, TGFBI, UBE2I	small molecule biochemistry, drug metabolism and tumor morphology	15

Network 2	ANXA2, CAPG, CLIC1, CPNE1, CSTB, DAP, EIF3B, GUK1, IFITM1, RPL34, RPS9, SLC7A11, SOX4, TBCD, TUFM, USP22	protein synthesis, cell signaling and interaction, and reproductive system function,	14

Network 3	ATP6V0B, BHLHE40, COTL1, CYBA, DDIT4, FTL, ITPA, LAPTM5, NEBL, NUDT1, RPS2, S100A11, SORD	genetic disorder, immunological disease and free radical scavenging	14

Network 4	ANXA2, ARPC1B, BUD31, GNPTG, GPATCH3, GTF3C1, NAP1L4, PHB, POLR2H, PUF60, UBA6, VKORC1, WDR36	gene expression, cellular development, cellular growth and proliferation	14

**Table 5:** 15 miRNAs and target genes involved in the cancer-related functions

Cancer. related Function	PLS predicted Target genes in the respective functions	The nos. of cancer related miRNAs associated with the mRNA targets
Cancer	ANXA2, BHLHE40, CANX, CAPG, COTL1, CYBA, DDIT4, FTL, GPX1, GSTP1, IFITM1, MIF, MMP11, OAZ1, ODC1, S100A11, SLC12A8, SLC7A11, SOX4 and UBE2I (20)	15

Tumorigenesis	ANXA2, BHLHE40, CANX, CAPG, COTL1, CYBA, DDIT4, FTL, GPX1, GSTP1, IFITM1, MIF, MMP11, NUDT1, OAZ1, ODC1, PRDX2, S100A11, SLC12A8, SLC7A11, SOX4 and UBE2I (22)	15

Carcinoma	ANXA2, BHLHE40, CANX, COTL1, CYBA, FTL, GSTP1, IFITM1, MMP11, OAZ1, ODC1, S100A11 and SLC7A11 (13)	12

Malignant tumor	ANXA2, BHLHE40, CANX, COTL1, CYBA, FTL, GPX1, GSTP1, IFITM1, MIF, MMP11, OAZ1, ODC1, S100A11, SLC7A11 and SOX4 (16)	12

Primary tumor	ANXA2, BHLHE40, CANX, COTL1, CYBA, FTL, GPX1, GSTP1, IFITM1, MIF, MMP11, OAZ1, ODC1, PRDX2, S100A11, SLC7A11 and SOX4 (17)	12

Angiogenesis	ANXA2, CANX and GPX1 (3)	8

**Table 6:** 15 miRNAs and target genes in the cancer-related canonical pathways

Cancer related canonical pathway	PLS predicted Target genes in the respective pathways	The nos. of cancer related miRNAs associated with the mRNA targets
Protein Ubiquitination Pathway	PSMA3, PSMB2, PSMB4, PSMC2, PSMC4, PSMD8, UBE2I, USP22(8)	14

Polyamine Regulation in Colon Cancer	OAZ1, ODC1, PSMA3, PSMB2, PSMB4, PSMC2, PSMC4, PSMD8(8)	15

Purine Metabolism	ATP6V0B, GUK1, ITPA, POLR2H, PSMC2, PSMC4 (6)	11

Circadian Rhythm Signaling	BHLHE40(1)	2

Hypoxia Signaling in the Cardiovascular System	UBE2I(1)	4

Cdc42 Signaling	ARPC1B, CDC42SE1(2)	4

Pyrimidine Metabolism	ITPA, POLR2H (2)	7

mTOR Signaling	DDIT4, EIF3B (2)	7

NRF2-mediated Oxidative Stress Response	FTL, GSTP1, PPIB(3)	7

Xenobiotic Metabolism Signaling	FTL, GSTP1(2)	7

### STEP 7. Combinatorial analysis with the aid of the PLS method and IPA pathway designer

For the above pathway analysis results from 31 down-regulated miRNAs and 71 up-regulated mRNAs in the previous step, we were interested in knowing which miRNAs were associated with the mRNAs grouped in the above cancer-related networks, functions and pathways predicted by our PLS regression method. With the aid of IPA pathway designer we expanded the above networks, functions and pathways generated by IPA (Figure 3, Tables 4, 5 and 6) by combining the PLS regression method and IPA pathway.

**Figure 4:**
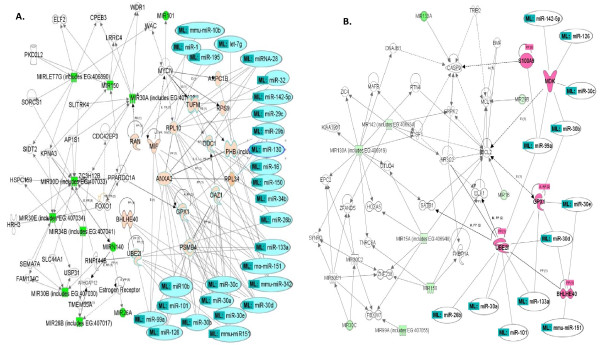
**Overlaid networks predicted by the PLS method and generated by IPA pathway designer**. **A. **Depicted is an overlaid miRNA-mediated network consisting of the top network 1 from Figure 3A on the left side of this network (green-colored miRNAs) and 27 miRNAs labeled with ML on the right that were significantly associated with the pink-colored mRNAs that were predicted by PLS. **B. **Depicted is an overlaid miRNA-mediated network consisting of the top network 2 from the Figure 3B on the left side of this network (green-colored miRNAs) and 12 miRNAs labeled with ML on the right that were significantly associated with the pink-colored mRNAsthat were predicted by PLS.

At first, we examined the two networks that were mediated by miRNAs and that were generated from the 31 down-regulated miRNAs (Figure 3A and 3B). With the aid of the IPA pathway designer, we connected miRNA-targets predicted by the PLS regression method with each associated miRNA labeled as my list (ML in Figure 4A and 4B). These miRNAs in ML were connected to the two networks (Figure 3A and 3B) through their predicted targets by IPA. The new expanded networks from Figure 3A and 3B are shown in Figure 4A and 4B. In Figure 4A, we found that 27 of 31 down-regulated miRNAs were associated with the miRNA-associated network through 15 genes (ANXA2, ARPC1B, BHLHE40, GPX1, MIF, OAZ1, ODC1, PHB, PSMB4, RAN, RPL10, RPL34, RPS9, TUFM, UBE2I) predicted by the PLS regression method. These mRNAs are all included in the list of predicted target in Table 2. It was interesting to see that the four remaining miRNAs (miR-100, rno-miR-140, miR15a and miR-26a) were grouped in this network by IPA and not connected through the above cancer-related target mRNAs by the pathway designer and hence two of them (miR-100 and miR-15a) were not included in Figure 4A. In this network, many of the cancer-affected target mRNAs were mainly regulated by the transcription factors MYCN and FOXO1 which play a vital role in cell death, apoptosis, proliferation and survival. Particularly MYCN was involved in the ERK/MAPK signaling pathway and cancer.

In a similar manner in Figure 4B, we found that 12 of 31-miRNAs were grouped with the miRNA-associated network through their predicted targets (BHLHE40, GPX1, MDK, S100A9 and UBE2I). These cancer-associated target mRNAs were connected to this network mainly through the interaction with apoptosis and cell death genes such as BCL2, CASP9 and ELK1 (Figure 4B).

Next we examined the expanded IPA results from 71 up-regulated mRNAs which were shown in the last column of Tables 4, 5 and 6. At first we wanted to know which of the 31 down-regulated miRNAs were associated with the top four networks through their target mRNAs predicted by PLS regression method. With the aid of IPA pathway designer, we found that 27 of the 31 down-regulated miRNAs were linked to one or more mRNA networks and 20 of them (let-7 g, miR-101, miR-126, miR-133a, miR-142-5p, miR-150, miR-15a, miR-26b, miR-28, miR-29b, miR-30a, miR-30b, miR-30c, miR-30d, miR-30e, miR-34b, miR-99a, mmu-miR-151, mmu-miR-342 and rno-miR-151) were involved in all of the top 4 networks. By restricting our attention to only the 15 cancer-associated miRNAs in the top four mRNA networks, we found that all 15 miRNAs were involved in network 1, all but miR-16 were in network 2, and all but miR-29c were in network 3 and 4, as shown in the fourth column of Table 4.

We also checked which miRNAs associated with the mRNA targets predicted by PLS regression method were linked to the cancer-related function. Through their targets, we found that 26 out of the 31 down-regulated miRNAs were associated with these targets and were in some ways related to cancer, 24 miRNAs were in the pathway of polyamine regulation in colon cancer and 12 miRNAs were involved in angiogenesis pathway. Again, we were interested in examining the 15 miRNAs directly linked with cancer-and their biological functions. We found that all 15 miRNAs were involved in cancer and tumorigenesis, 12 of them (all except miR-101, miR-15a and miR-29c) were in carcinoma, malignant tumor and primary tumor and 8 of them (all except let-7 g, miR-101, miR-150, miR-15a, miR-16, miR-29c and miR-342) were in angiogenesis as were shown in the last column of Table 5.

Furthermore, we examined which associated miRNAs among the 15 cancer-related miRNAs were involved in the canonical pathways associated with cancer. We found that 14 miRNAs were associated with protein ubiquitination pathway, all 15 miRNAs were in polyamine regulation in colon cancer, 11 miRNAs were associated with purine metabolism, 2 miRNAs were associated with circadian rhythm signaling, 4 miRNAs each were associated with hypoxia signaling in cardiovascular system and cdc42 signaling, and 7 miRNAs each were associated with pyrimidine metabolism, mTOR signaling or NRF2-mediated oxidative stress response or xenobiotic metabolism signaling along and their targets are mentioned in the last column of Table 6.

Overall, it clearly showed that these cancer-related miRNAs may mediate many biological functions and pathways by regulating the target genes predicted by our integrative and computational method.

### STEP 8. Estimating false detection rate for PLS based target prediction

**Figure 5:**
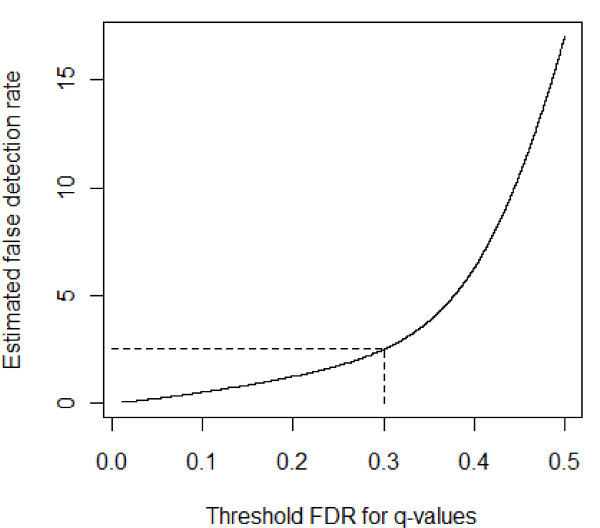
**Estimated false detection rates for different thresholds**. Various thresholds for the q-values are on the x-axis. The estimated false detection rate corresponding to each threshold is given on the y-axis. For the selected threshold of 0.3, the estimated false detection rate is 2.50%.

In order to determine the false detection rate of our mRNA target prediction method using PLS regression we used a simulation experiment. As defined in [22], the false detection rate is the percentage of detected significant miRNA-mRNA pairs derived as output from the PLS method out of the total possible pairs that would have the same or better associations by chance. Based on this procedure, the estimated false detection rate for the threshold FDR of 0.3 was 2.50%. We then reported the estimated false detection rates for many values of the thresholds simultaneously, and the estimated false detection rate is plotted as a function of the threshold in Figure 5.

We also compared the PLS regression method with a simpler method based only on the correlations for all miRNA-mRNA pairs. A correlation threshold of -0.750 corresponded to an estimated false detection rate of 2.50%. The PLS based method was more sensitive in detecting targets when applied to the actual data set; only 33 miRNA-mRNA connections were declared to be significant based on the correlation analysis with a threshold -0.750, whereas 155 connections were significant for the FDR threshold of 0.3.

## Discussion

### Integrative computational modeling of miRNA targets using PLS

Although there are many computational algorithms for predicting miRNA targets, they vary widely with regards to specific targets identified. The relative usefulness of these algorithms is strengthened if there are some agreements in their conclusions. However, that is usually not the case among the existing algorithms. Additionally, the existing algorithms depend on the sequence complementarity of the miRNA-mRNA pairs. So, more complex algorithms using joint expressions of miRNA and mRNA may be useful to predict miRNA targets. In this study we developed an approach for predicting miRNA-mRNA associations based upon a statistical analysis using unpaired t-tests, PLS regression based association scores and the known inverse relationship between certain mRNAs and miRNAs.

Our efforts focused on a set of 31 significantly down-regulated miRNAs and a set of 71 significantly up-regulated mRNAs identified by the t-test for colon cancer vs. the control samples after multiple hypotheses corrections. We selected these miRNAs and mRNAs based on their inverse relationship for our integrative analysis. Using miRNAs as independent variables and mRNAs as dependent variables in our PLS regression method we identified significant miRNA-mRNA associations and in turn predicted mRNA targets for individual miRNAs (Table 1). When comparing these to miRNA-targets predicted by TargetScan 5.1 and miRanda, we found on average about 16.5% and 11.0% of our predicted targets matched those predicted by these two programs, respectively. Furthermore, some of our predicted target genes lie within the union or intersection of the other two target prediction programs. It seems likely that the miRNA target genes that lie within such intersections could be more accurate, although this claim remains to be evaluated further, experimentally. Interestingly, many of the targets predicted by our methods are not predicted by the other prediction algorithms and thus our approach provides possible novel targets that need further investigation for relevance to colorectal cancer {Step 7}. We estimated the false detection rate of the proposed method through a simulation study by sampling from the real data under the null model. A limitation of the present study is that we are unable to compute statistical performance measures such as the false discovery rate since there does not exist any realistic simulator currently for generating miRNA and mRNA datasets simultaneously with controlled biological interactions between them.

We have also compared our computational method of target prediction with another computational method of target prediction by association resulting from pairwise simple correlations between all 31 the up-regulated miRNAs and 71 down-regulated mRNAs. For the same dataset using the same threshold we get many more targets and so the PLS based method proved to be more sensitive than just correlation based target prediction described in Step 8 in the Results section.

## Conclusion

In summary, we have developed a bioinformatics computational approach for the analysis of miRNA and mRNA expression profiles derived from the same set of cancerous tissue samples to infer the molecular mechanism for the colon cancer etiology. This analysis provides an alternative computational approach to identify putative miRNA targets and reverse engineer the miRNA networks with their mRNA targets. We have developed a PLS modeling approach to take advantage of the known inverse relationships between some miRNAs and their target mRNAs. This approach would certainly have to be modified for those situations where such a relationship does not exist. Our analysis resulted in 3 major outcomes. 1) First, several of the predicted miRNA targets identified with our approach have also been predicted by other target prediction algorithms, which provide some support for the development of our methods. 2) The PLS regression approach adopted here has resulted in the recognition of novel miRNA-mRNA sets (in turn miRNA targets). These putative data-sets could provide better understanding of the underlined molecular mechanism of colon cancer or any other complex disease and identify novel therapeutic targets upon experimental validations. 3) An *in silico *analysis of the biological significance of the results obtained with our PLS approach and pathway analysis indicates that processes related to protein degradation and cell death perhaps initiated by oxidative stress are likely involved in colorectal cancer. This fact has been concluded by other experimental studies [45]. Overall, it clearly shows that these down-regulated miRNAs for colon cancer can most likely be involved in many altered biological functions along with their targets. The utility of our approach and its possible applicability to other disease states awaits further experimental validation.

## Methods

### miRNA and mRNA microarray data and gene expression profile analysis

The miRNA and mRNA microarray data sets for both human colon tumors and normal tissues used here were obtained from the Broad Institute and downloaded from their web-accessible database: http://www.broadinstitute.org/cgi-bin/cancer/datasets.cgi [20]. The data sets originally consisted of five normal human tissues and 10 human colon tumors for both miRNA and mRNA. However, only four of the normal tissue samples and seven of the colon tumor samples passed quality control criteria [46]. The mRNA expression values were obtained using Affymetrix GENECHIP analysis software (after hybridization to Affymetrix oligonucleotide microarrays Hu6800 and Hu35KsubA) containing a total of 16,063 probes [46]. The miRNA median fluorescence intensity values were measured by using a new, bead-based flow cytometric miRNA expression profiling method. All data were filtered by minimum value 32 (values less than 32 were considered as a background value according to the reference) and log_2 _transformed [20].

The miRNA and mRNA expression data sets for colon cancer patients were loaded into GeneSpring GX 10.0 (Agilent Technologies). An unpaired t-test was used to compare miRNA and mRNA expressions for normal and tumor tissue samples. The p-values obtained were then adjusted with the aid of the Benjamini-Hochberg false discovery rate (FDR) [28] correction method. The differentially expressed miRNAs or mRNAs with q-values less than or equal to 10% and the absolute fold changes greater than or equal to 1.2 were determined to be significant.

### miRNA target prediction using partial least square (PLS) method and bootstrapping

An algorithmic version of the whole procedure is provided in Figure 1. A detailed description of the PLS regression method used in this paper was previously reported in [23] and [27]. Briefly, consider a data set with *n *observations of *p *miRNAs (predictor variables) and *m *mRNAs (response variables). Let *Y *= (*Y*_1*i*_,*Y*_2*i*_, ...,*Y_ni_*)' be the column vector of observed values for the *i*th mRNA;*i *= 1,2, ... ,*m*, and let *X_j _*= (*X*_1*j*_, *X*_2*j*_, ..., *X_nj_*)' be the column vector of observed values for the *j*th miRNA for *j *= 1,2, ...,*p*. For each mRNA *Y_i_*, PLS fits a regression model of the form

where *e_i _*is an *n*-dimensional vector of residuals and  is the k-th latent variable amongst the set of all orthogonal latent variables (*l*<<<*p*), all of which are linear combinations of the covariates. All of the original variables are standardized before doing PLS.

Using ordinary least squares leads to

and then the coefficients associated with the original covariates can be obtained from the coefficients of the latent variables.

Specifically, after *q *latent factors are found by selecting the number of component as 3, we calculated the scaled score coefficient . Then the score coefficients  and intercept  can be obtained by transforming  back to the original scale as follows ,

and

. These coefficients are called the PLS scores, and these scores are defined as the associations between each response variable (mRNA) and the miRNA covariate  for a specific *i *and *j*.

### Statistical test of the significance of the association between miRNA and mRNA (*β_ij_*)

In order to test the significance of the association between miRNA and mRNA, we tested the null hypothesis is H_0_: *β_ij _*= 0, (which implies there is no significant association between a specified miRNA and mRNA) versus the alternative H_a_: *β_ij_*< 0( which implies there is an inverse association between them). As we do not know the asymptotic distribution of such associations we used a bootstrap re-sampling scheme to construct the sampling distribution of the score *β_ij _*for testing the hypothesis.

We ran PLS after removing the *j*th miRNA gene, to generate new *β_ij _*and we compute the residual of the model which is the estimate of the error *e_i_*.

After the residuals are computed, bootstrapping residuals  are generated by simple random sampling with replacement of the components *e*_*i*1_,*e*_*i*2_,...,*e_in _*of the residual vector *e_i_*. Then the bootstrapped  data can be found by the original regression equation:

where the parameters hold their usual meanings. We then run PLS with the new  and original full set of miRNAs, and store the bootstrapped PLS based score . We did this bootstrapping step 1000 times for each gene and calculate the proportion of times when the negative bootstrapped association scores were smaller than negative observed association score . These proportions are the estimated p-values of the tests. The Benjamini & Hochberg FDR [28] then is used to calculate q-values. A threshold FDR was arbitrarily chosen as 0.3 since the number of variables in this test was small after having identified the inverse relationship between miRNA and mRNA. The associations for which the q-values were less than equal to the FDR cutoff were deemed to be significant.

### Simulation Experiment

A simulation method similar in concept to the one proposed in [22] was used to estimate the false detection rate of possible targets using PLS regression for a specific threshold of FDR. The definition of the false detection rate was provided before in Step 8 of the Results section, as described on page 4 of [22]. Note that the concept of false detection rate refers to the simulation-based estimate of the percentage of detected significant miRNA-mRNA pairs derived by chance, and when using this concept, we refer to it in full as false detection rate so as not to confuse it with the abbreviation FDR which we have already reserved for the more commonly-used false discovery rate [28]. While we still use the FDR to control false positives for our PLS method, we use the false detection rate for this simulation to determine equivalent thresholds for our method and for a simple correlation method which does not choose its threshold based on FDR.

To generate a simulated data set with *n *"unassociated" observations of *p *miRNAs and *m *mRNAs, we randomly permuted the order of the *n *observations for both the miRNA genes and the mRNA genes so that none of the miRNA arrays corresponded to its true mRNA array. Specifically, we randomly permuted the labels of both arrays and rejected any generated data sets for which the new labels matched. Therefore, since the miRNAs do not correspond to the mRNAs in the simulated data set, any low q-values only occur by chance. For the simulated data set, we then compute the number of false positives by counting the number of q-values which fell below the threshold. The process was repeated for 1000 simulated data sets and the estimated false detection rate was computed by dividing the total number of false positives for the 1000 data sets by the overall total number of miRNA-mRNA pairs for the 1000 data sets.

We also compared the PLS regression with a simpler method based only on the correlations for all miRNA-mRNA pairs. In the correlation method, we declare that a miRNA-mRNA pair is significant if the corresponding pairwise correlation fell below a certain threshold. To compare the sensitivity of the methods, we used the same simulation method described above, and we chose the threshold for which the estimated false detection rate was the same as that found for the PLS regression method.

### Biological function and pathway analysis

To obtain gene ontology (GO) terms for each miRNA, we used Pathway Studio software (Ariadne Genomics Inc., Rockville, MD, USA). This software enabled an enrichment analysis of expressed genes for GO terms using the differentially expressed miRNA target genes with respect to the category of biological process. The GO terms were selected by significant p-value and cancer-associated biological functions.

Using Ingenuity Pathway Analysis System and pathway designer (IPA 8.5, Ingenuity Systems, Edwood, CA, USA), we performed more detailed functional analysis to identify miRNA-mediated, cancer-related and statistically significant networks, biological functions and canonical signaling pathways for both differentially expressed miRNAs and mRNAs.

## Competing interests

The authors declare that they have no competing interests.

## Authors' contributions

XL conducted the computational analysis and pathway analysis. RG conducted the simulation. JY, RG and SD helped with PLS coding in R. NGFC helped with the biological pathway analysis and interpretation. SD designed and supervised the project. XL, RG, and SD wrote manuscript. All authors read and approved the final manuscript.

## Pre-publication history

The pre-publication history for this paper can be accessed here:

http://www.biomedcentral.com/1755-8794/4/44/prepub

## Supplementary Material

Additional file 1 **Differentially expressed miRNAs in human colon tissues**. Supplemental Table 2 (S2): This file is a tab-delimited text file and contains 2 up-regulated and 31 down-regulated miRNAs with FDR corrected p-value less than 0.1 and absolute fold changes greater than 1.2. It also provides detailed information about each miRNA such as its name, expression values and description. Click here for file

Additional file 2 **Differentially expressed mRNAs in human colon tissues**. Supplemental Table 1 (S1): This file is a tab-delimited text file and contains 73 up-regulated and 63 down-regulated mRNAs with FDR corrected p-value less than 0.1 and absolute fold changes greater than 1.2 It also provides detailed information about each mRNA such as its name, expression values and description. Click here for file
